# Machine Learning-Driven Advances in Perovskite Materials and Solar Cells

**DOI:** 10.3390/nano16140898

**Published:** 2026-07-22

**Authors:** Jun Ren, Xiangshun Geng, Shangjian Liu, Qinghua Liu, Shuoying Li, Tian-Ling Ren

**Affiliations:** 1 School of Basic Education, Beijing Information Technology College, Beijing 100018, China; renj@bitc.edu.cn (J.R.); liuqinghua@bitc.edu.cn (Q.L.); lishuoying060921@163.com (S.L.); 2 School of Integrated Circuit, Tsinghua University, Beijing 100084, China; liushangjian2018@gmail.com; 3 Beijing National Research Center for Information Science and Technology (BNRist), Tsinghua University, Beijing 100084, China

**Keywords:** machine learning, perovskite solar cell, renewable energy technology, material screening, intelligent system integration

## Abstract

Driven by advances in renewable energy technologies, research on perovskite optoelectronics has advanced rapidly across material exploration, device engineering, and intelligent integrated systems. Conventional trial-and-error experiments face inherent constraints in precisely regulating perovskite chemical compositions and microstructures, as well as in mitigating degradation in perovskite solar cells (PSCs). Artificial intelligence (AI) and the Internet of Things (IoT) have emerged as powerful tools for material discovery, synthetic condition design, and the prediction of perovskite fundamental properties and device outputs. This review systematically summarizes recent advances in machine learning (ML) implementations for PSC research, covering molecular-scale material screening, synthetic parameter optimization, performance forecasting, device architecture design, and system performance evaluation. We further elaborate on key obstacles hindering ML-assisted perovskite development, including insufficient operational stability, barriers to large-scale fabrication, and limited computational efficiency. Last, we outline promising research avenues and highlight the transformative capacity of ML to advance high-performance, manufacturable perovskite optoelectronic devices.

## 1. Introduction

**Figure 1:**
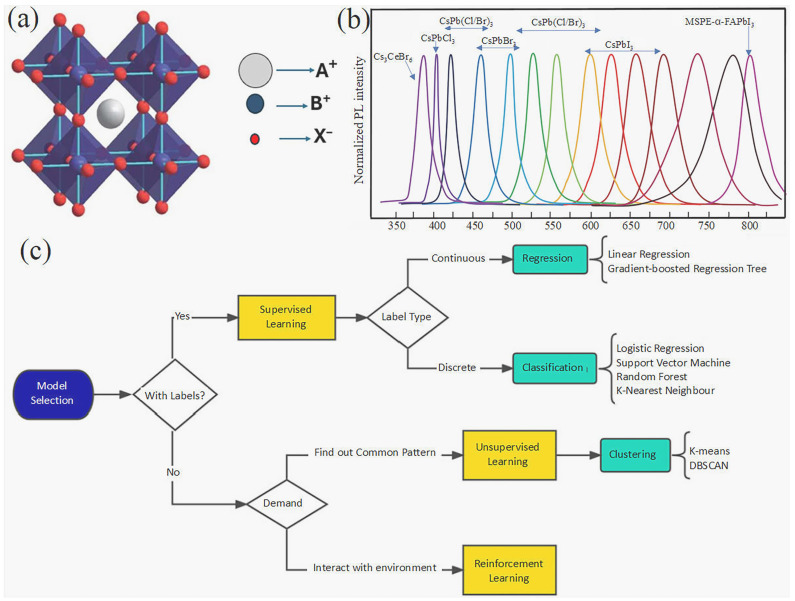
(**a**) Schematic crystal structures of ABX_3_ perovskite crystal. Reprinted from Ref. [6]. (**b**) The normalized photoluminescence spectra of CsPbX_3_-based perovskites. Reprinted from Ref. [8]. (**c**) Flowchart for ML model selection in common PSC research, particularly in materials discovery and optoelectronic properties research. Reprinted from Ref. [38].

Metal-halide perovskites have attracted extensive attention in optoelectronic and photovoltaic applications because of their high absorption coefficients, tunable bandgaps, long carrier diffusion lengths, long carrier lifetimes, defect tolerance, and compatibility with solution-based fabrication processes [1,2,3,4,5]. The general formula of metal-halide perovskites is ABX_3_, where A is typically an organic or inorganic monovalent cation, B is a divalent metal cation, and X is a halide anion, as illustrated in Figure 1a [6]. By tailoring the A-site cation, B-site metal, and halide composition, the optical and electronic properties of perovskites can be adjusted over a wide range, making them promising candidates for light-emitting diodes, photodetectors, lasers, and solar cells. Studies have shown that by varying the composition in all-inorganic halide perovskites, the emission wavelength can be continuously modulated across the entire visible spectrum by varying the X-site halide anion components, as shown in Figure 1b [7,8]. Among these applications, perovskite solar cells (PSCs) have shown particularly rapid progress since the first MAPbI_3_-based solar cells were reported in 2009. Their power conversion efficiency (PCE) has increased dramatically over the past decade, with certified single-junction PSC efficiencies now exceeding 26% and perovskite/silicon tandem solar cells approaching or exceeding the efficiency range of state-of-the-art crystalline silicon photovoltaics [9,10,11]. These advances demonstrate the great potential of PSCs as next-generation photovoltaic technologies.

Despite this rapid progress, the development of high-performance and stable PSCs still relies heavily on empirical optimization. The performance of PSCs is governed by a complex combination of factors, including perovskite composition, precursor chemistry, solvent engineering, crystallization dynamics, defect passivation, charge-transport layers, interface energetics, device architecture, encapsulation, and environmental operating conditions [12,13,14,15,16]. Conventional trial-and-error experimentation can be effective for local optimization, but it is often time-consuming, material-intensive, and difficult to generalize across different material systems and processing conditions. High-throughput experimental platforms, such as automated synthesis and microfluidic screening systems, can improve experimental efficiency [15], but their implementation often requires specialized equipment, substantial maintenance costs, and standardized data workflows. These limitations motivate the development of data-driven strategies that can accelerate material screening, process optimization, and device design.

Physics-based computational methods, including density functional theory (DFT), molecular dynamics (MDs), Monte Carlo simulations, and finite element modeling, have played indispensable roles in perovskite research [17,18,19,20,21,22,23,24]. DFT is particularly important for understanding electronic structures, defect formation, ion migration, and structure-property relationships, while MDs and finite element methods provide insights into dynamic processes and device-level physical behavior. However, the direct application of these methods to large compositional spaces, complex device architectures, or manufacturing-scale process optimization can be limited by computational cost, system size, timescale, and the difficulty of incorporating diverse experimental variables. For example, Xu et al. spent 72 h simulating the phase separation behavior of mixed-halide perovskites using DFT, with a total cost exceeding $5 × 10^4^ [25]. Wang et al. demonstrated the development of full-visible-spectrum CsPbX_3_ quantum dots via conventional trial-and-error. To cover the 400–700 nm emission range, 320 experiments were conducted over 14 months, consuming precursor materials worth approximately $8 × 10^4^ and highlighting the inefficiency of traditional methods [26]. Machine learning (ML), as a core branch of artificial intelligence, aims to identify patterns and predictive relationships from data. Rather than replacing first-principles or physics-based simulations, ML can serve as a complementary tool that learns structure–composition–processing–property relationships from experimental, computational, and literature-derived datasets. When properly validated, ML models can help prioritize candidate materials, identify key descriptors, construct surrogate models, and guide subsequent DFT calculations, device simulations, or experimental verification.

In perovskite research, input data can be obtained from published literature, open databases, high-throughput experiments, or high-throughput calculations performed using tools such as Materials Studio, the Vienna Ab initio Simulation Package (VASP), LAMMPS, and Car–Parrinello molecular dynamics [27,28,29,30,31,32]. Depending on the task, supervised learning methods, including random forest (RF), support vector machine, kernel ridge regression (KRR), gradient boosting models, and neural networks, have been used to predict bandgaps, formation energies, stability indicators, PCE, and processing-performance relationships [33,34]. Unsupervised learning methods can further help classify materials, discover hidden patterns, and identify clusters in high-dimensional datasets without predefined labels [35,36,37,38]. As schematically summarized in Figure 1c, a typical ML workflow includes data collection, descriptor construction, model training, validation, prediction, and experimental or computational feedback. Table 1 provides an overview of recent studies on ML applications in predicting and optimizing bandgap, formability, and stability of perovskite materials. It highlights various ML techniques employed for objectives ranging from predicting PCE and stability to optimizing material properties and synthesis processes [28,39,40,41,42,43,44,45,46,47,48,49]. Specifically, during the materials discovery stage, models such as random forest, support vector machine, and logistic regression are widely used. These algorithms efficiently process high-dimensional and complex datasets, thereby accelerating the identification of new materials [44,45]. During device performance optimization, RF, GBM, and KRR are commonly adopted to predict key performance indicators, including PCE, bandgap, and doping concentration. For process optimization, ML models are applied to fine-tune experimental parameters, such as temperature and solution concentration, thus improving manufacturing efficiency. Finally, in system integration, the goal is to optimize the overall performance of photovoltaic systems under varying environmental conditions. The reliability of such workflows depends strongly on dataset quality, descriptor selection, model interpretability, uncertainty quantification, and validation beyond the training domain.

**Table 1:** Overview of recent studies on ML applications of bandgap, formability, and stability of perovskite materials.

Year	ML Technique	Objective	Results	Ref.
2025	Machine learning interatomic potential	Structure, band gap and ion migration energies	The integration of ab initio and ML methods yields simulated structure, band gap and ion migration energies consistent with experimental measurements. Partial substitution of Pb site by Sn^2+^, Ba^2+^, Cu^2+^ barely inhibits iodide ion migration, offering key guidance for subsequent doping research. The ML interatomic potential enables large-scale ~80 ns simulations on pure and Sn-doped γ-CsPbI_3_.	[28]
2023	GPR	Formability	Identified key structural descriptors and validated MLFF methods for CH_3_NH_3_PbBr_3_, revealing symmetry-breaking effects and successfully simulating large-scale trajectories for CsPbI_3._	[39]
2023	VAE, tVAE, cVAE	Bandgap	Identify primary variability in PL controlled by bandgap compositional dependence; secondary variability includes phase separation. VAEs effectively disentangle latent factors of variability, providing insights into bandgap shifts and stability mechanisms.	[40]
2021	GBR, SVR, BPANN, and RF	Hydrogen production rate and bandgap	Screened fourteen potential perovskite photocatalysts with average hydrogen yield 6.4% higher than the highest value in the training dataset, and developed a web server to predict hydrogen yield and bandgap of ABO_3_ perovskite materials.	[41]
2020	ANN	Bandgap	Achieve significant prediction accuracy for band gaps, demonstrating that machine learning can effectively guide the discovery of new photovoltaic materials with optimal properties	[42]
2019	LR, KNN, SVR, RF, and ANN	Bandgap, PCE	Achieved RMSE of 0.060 eV and r value of 0.97 for bandgap prediction using ANN. PCE prediction using ANN showed RMSE of 3.23% and r value of 0.80 with true bandgap, and RMSE of 3.27% and r value of 0.72 with predicted bandgap.	[43]
2022	GBR, SHapley additive explanation(SHAP)	PCE, bandgap	Identify four lead-free perovskite candidates: (CH_3_NH_3_)_2_AgGaBr_6_, (CH_3_NH_3_)_2_AgInBr_6_, (C_2_NH_6_)_2_AgInBr_6_, and (CH_3_NH_3_)_2_AgAlBr_6_, with predicted efficiencies of 20.6%, 19.9%, 27.6%, and suitable thermal conductivities, and band gaps for photovoltaic applications	[46]
2025	Symbolically Encoded Neural Network(SENN)	Bandgap, composition	SENN exhibits superior prediction accuracy and model stability compared with conventional machine learning algorithms, possessing excellent generalization performance. The model serves as a quantitative analysis tool to reveal the intrinsic relationship between perovskite composition and bandgap.	[47]
2024	XGBoost, Matminer, Shapley	Bandgap	A dataset of 447 ABX_3_ inorganic halide perovskites is constructed with Matminer-derived descriptors. The XGBoost classifier achieves 95% accuracy in identifying narrow-bandgap perovskites.	[48]
2023	RF, Genetic algorithm-based symbolic regression, Natural language processing	Bandgap, stability, formation energy	The multi-objective DFT and RF screening framework filter out three candidate 2D lead-free halide perovskites with matching band gap and good stability. Molecular dynamics simulations verify the thermodynamic stability of the three candidates, calculated SLME values confirm photovoltaic suitability.	[49]

Recent years have witnessed a growing number of studies applying ML to perovskite materials and PSCs, including materials discovery, property prediction, stability assessment, device optimization, scalable processing, and commercialization-related monitoring. Nevertheless, important challenges remain. Many reported ML models are trained on small, heterogeneous, or biased datasets; validation is often limited to internal cross-validation; uncertainty and out-of-distribution behavior are not always reported; and the distinction between experimentally measured, computationally simulated, ML-predicted, and certified performance values is sometimes unclear. These issues are particularly important for PSCs because laboratory-scale efficiency, long-term operational stability, scalable manufacturing, and module-level reliability do not necessarily improve simultaneously.

Compared with previous reviews that primarily summarized representative ML algorithms and their applications in PSCs [50,51,52,53], this review emphasizes the scientific confidence, validation level, and practical translation of ML-driven PSC research. We critically discuss how ML has been used for perovskite material screening, property prediction, device performance optimization, stability analysis, and manufacturing-related monitoring. Particular attention is paid to dataset quality, model generalization, interpretability, uncertainty quantification, the complementary relationship between ML and physics-based simulations, and the transition from prediction to experimental validation. Finally, we outline a roadmap for reliable ML-assisted PSC development, including standardized datasets, benchmark protocols, closed-loop optimization, stability-oriented prediction, scalable manufacturing, and industrial deployment. Following this perspective, this review first discusses ML-guided prediction of intrinsic perovskite properties, then examines high-throughput composition screening, device-level performance optimization, stability assessment, and scalable manufacturing, and finally summarizes the remaining challenges and future perspectives for reliable ML-assisted PSC development.

## 2. Research Methodology

In recent years, the integration of ML with perovskite research has become a prominent avenue driven by its powerful data analysis capabilities. Compared with the limitations of time-consuming, labor-intensive manual efforts, ML accelerates big data filtering through its strong classification and regression capabilities, rapid computation speed, and ability to reduce false positives. Furthermore, combining ML with device fabrication offers a promising strategy for optimizing perovskite photovoltaic components. In this review, we offer a brief summary of recent advances in ML-driven perovskite materials and PSC devices, mainly in the prediction of material structure and performance, material screening and optimization, and experimental design and guidance. This review shows great potential in terms of efficiency.

**Figure 2:**
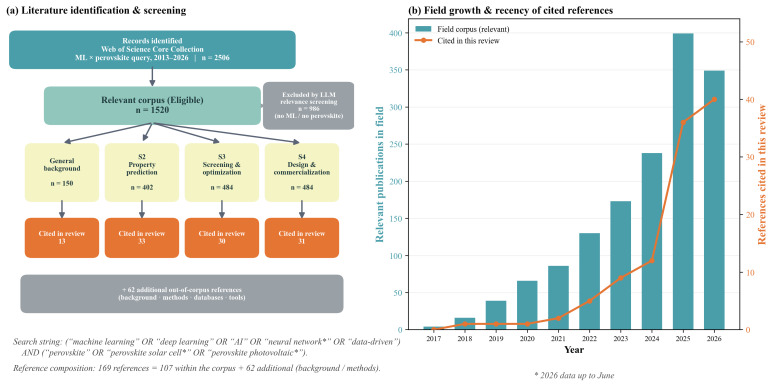
Bibliometric overview of the literature basis of this review. (**a**) Workflow of literature identification and screening. A Web of Science Core Collection search combining machine learning and perovskite terms returned 2506 unique records from 2013 to 2026; large language model (LLM)-assisted relevance screening removed 986 records that did not genuinely address the machine learning × perovskite intersection, leaving 1520 relevant publications. These were assigned to four thematic groups according to the structure of this review (S1, general background; S2, property prediction; S3, screening & optimization; S4, design & commercialization). Representative papers selected from each group, together with additional background and methodological references, constitute the references cited in this review. (**b**) Annual number of relevant publications in the field (bars, left axis), showing the near-exponential growth of machine learning-driven perovskite research, and the publication-year distribution of the references cited in this review (line, right axis). 2026 data up to June.

To construct a representative and up-to-date literature basis for this review, we performed a structured search of the Web of Science Core Collection using a two-block query that intersects machine learning terms (“machine learning”, “deep learning”, “AI”, “neural network”, “data-driven”) with perovskite terms (“perovskite”, “perovskite solar cell”, “perovskite photovoltaic”). This search yielded 2506 unique records (Figure 2a).

To systematically remove out-of-scope records, we employed an LLM-assisted relevance screening procedure using the Gemini 3.1 Flash Lite model (Google), with the temperature set to 0 for deterministic output. Each of the 2506 records was screened independently in a single API call. For every paper, the model received a structured prompt consisting of (i) a system prompt that defined the review’s thematic scope and instructed the model to judge relevance based on scientific understanding of the content rather than rigid keyword matching, and (ii) a user prompt containing the paper’s title, author keywords, KeyWords Plus, and abstract. A paper was classified as “relevant” only if it substantively involved both machine learning/AI/data-driven methods and perovskite materials or perovskite-based photovoltaic/optoelectronic devices. For each relevant paper, the model further assigned it to the single best-fitting thematic section of this review. This step removed 986 out-of-scope records, leaving a relevant corpus of 1520 publications. To validate the consistency between the LLM-assisted and manual screening, we randomly sampled 25 papers from the relevant pool (*n* = 1520) and 25 from the excluded pool (*n* = 986) and had a human reviewer independently evaluate them against the same inclusion/exclusion criteria. The inter-rater agreement was 96.0% (48/50; Cohen’s κ = 0.920) between the automated and manual judgments.

The relevant corpus was then organized into four thematic groups mirroring the structure of this review: general background, prediction of intrinsic properties, high-throughput screening and composition optimization, and device design, stability, and commercialization. From each group, we selected representative studies for in-depth discussion, and complemented them with additional background and methodological references, together forming the references cited in this review. The temporal distribution of the corpus confirms that machine learning-driven perovskite research is a rapidly growing field: the annual number of relevant publications grows almost exponentially (Figure 2b). The publication years of the references cited in this review closely track this trend; in particular, a large fraction of our citations are drawn from work published in 2025 and 2026, ensuring that the review reflects the most recent advances.

## 3. ML-Guided Prediction of Perovskite Intrinsic Properties

**Figure 3:**
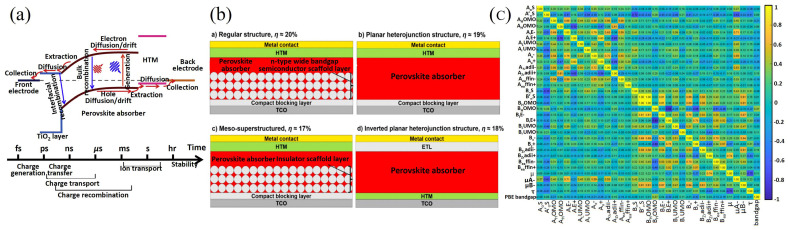
(**a**) Possible dynamic processes and their corresponding time scales within the cell. Reprinted from Ref. [54]. (**b**) Different structural configurations of PSCs. Reprinted with permission from Ref. [55]. Copyright 2026 American Chemical Society. (**c**) Pearson correlation analysis of bandgap characteristics. Reprinted from Ref. [56].

The intrinsic properties of perovskite materials, including bandgap, formation energy, carrier mobility, defect tolerance, structural stability, and dimensionality, determine their suitability for photovoltaic and optoelectronic applications [4,5,6,7,8,9,10,11,12,13,14,15,16,17,18,19,20,21,22,23,24,25,26,27,28,29,30,31,32,33,34,35,36,37,38,39,40,41,42,43,44,45]. The photogenerated carrier dynamics mainly involve stages of generation, relaxation, diffusion, interfacial recombination, and extraction/collection over timescales ranging from femtoseconds to milliseconds, as shown in Figure 3a [54]. These transient processes determine the optoelectronic properties of semiconductors and are directly related to the final performance of the devices. Four typical PSC device structures exist, as shown in Figure 3b: regular (mesoporous TiO_2_ ETL with a compact blocking layer), planar heterojunction (non-porous ETL), meso-superstructured (Al_2_O_3_ scaffold), and inverted (ETL and HTL positions swapped) [55]. The regular and planar structures follow an n-i-p configuration, while the inverted structure is p-i-n. A major challenge for PSC is stability. Most reports focus on macroscopic efficiency and I-V parameters over time, neglecting microstructural changes. These structures are indispensable for device composition and significantly influence the photogenerated carrier dynamics. Meanwhile, the roles of other device structures in affecting device performance, as well as the underlying physical mechanisms, have not yet been comprehensively discussed.

Bandgap prediction is one of the most widely studied ML tasks in perovskite materials because bandgap directly affects light absorption, open-circuit voltage, and photovoltaic performance. Compared with purely empirical rules, ML models can incorporate nonlinear relationships among composition, crystal structure, and electronic descriptors. For example, Wang et al. developed a bandgap prediction framework for lead-free double perovskites using a dataset containing 2367 entries collected from the Materials Project and literature, as shown in Figure 3c [56]. Several models, including multilayer perceptron (MLP), deep ensemble learning, physics-informed neural networks, and Transformer-based models, were compared. The MLP model achieved strong predictive performance, with a test-set R^2^ of 0.9311, MAE of 0.1915 eV, MSE of 0.0975 eV^2^, and RMSE of 0.3122 eV. In addition, 98% of the test samples showed prediction errors below 0.4 eV. SHAP analysis identified electronic-structure-related descriptors, such as B-site HOMO-related and A-site LUMO-related features, as important contributors to bandgap prediction. This study illustrates that interpretable ML can not only accelerate bandgap screening but also reveal chemically meaningful descriptors. Because the dataset combines database-derived and literature-derived values, further external validation on newly synthesized compounds would be necessary to evaluate the model’s transferability beyond the training domain.

Accurate prediction of these properties is therefore a central task in perovskite materials discovery. Traditionally, these properties have been investigated through experimental characterization and physics-based calculations, particularly DFT. DFT provides valuable insights into electronic structures, defect formation, ion migration, and structure-property relationships. However, exhaustive DFT calculations over large compositional spaces remain computationally expensive, especially for mixed-cation, mixed-halide, low-dimensional, defect-containing, or dynamically disordered perovskite systems. ML offers a complementary strategy by learning statistical relationships between material descriptors and target properties from experimental, computational, and literature-derived datasets. When properly trained and validated, ML models can rapidly screen large candidate spaces and identify promising compositions for subsequent DFT calculation or experimental verification. In this section, we focus on ML-assisted prediction of intrinsic material properties, including bandgap, carrier mobility, frontier orbital energy levels, structural stability, and phase dimensionality, while device-level performance optimization and manufacturing-related applications are discussed in subsequent sections.

A typical ML workflow for perovskite property prediction involves data collection, descriptor construction, model training, validation, interpretation, and candidate screening. Data can be obtained from experimental reports, high-throughput calculations, public materials databases, and manually curated literature datasets [57,58,59,60,61,62,63,64,65,66,67,68,69]. Common descriptors include elemental properties, ionic radius, electronegativity, atomic number, oxidation state, tolerance factor, octahedral factor, formation energy, orbital energy levels, lattice parameters, bond length, bond angle, and structural dimensionality. However, the reliability of ML predictions strongly depends on the quality, representativeness, and consistency of the underlying datasets. In perovskite research, datasets are often limited by small sample size, nonuniform experimental conditions, publication bias, incomplete reporting of processing parameters, and inconsistencies between experimental and computational labels. Therefore, data curation, feature selection, external validation, and uncertainty quantification are essential for building reliable ML models rather than merely achieving high internal cross-validation scores.

Carrier mobility is another important intrinsic property because it influences charge transport, recombination, and device efficiency. Nukunudompanich et al. proposed a neural network model with an additive kernel regression activation function for predicting bandgap and carrier mobility in lead-free perovskites [70]. By mapping approximately 40 descriptors, including atomic radius and electronegativity, into a high-dimensional nonlinear feature space, the model improved its ability to capture complex composition–property relationships. The model predicted a bandgap of 1.28 eV for MASnI_3_, close to the experimentally reported value of 1.30 eV, and a carrier mobility of 312 cm^2^ V^−1^ s^−1^, close to the reported value of 298 cm^2^ V^−1^ s^−1^. The same model was further used to screen Cs_2_TiBr_6_ as a candidate lead-free perovskite, with a theoretically estimated PCE of 18.7%, which is a fourfold increase over the traditional trial-and-error method. This example demonstrates the potential of ML for identifying environmentally benign perovskite candidates. It should be noted that the predicted device efficiency should be treated as a theoretical or model-derived value rather than as experimentally certified photovoltaic performance.

ML has also been used to predict frontier orbital energy levels and molecular descriptors relevant to perovskite precursor design, charge-transport materials, and organic components in hybrid perovskites. Ahmad et al. rapidly evaluated more than 40 regression models using the Lazy Predict Python library to screen organic compounds for perovskite-related applications [71]. Among the tested models, RF showed the best overall predictive performance and was selected for further analysis. HOMO and LUMO energy levels are particularly important because they affect energy-level alignment, charge extraction, and recombination losses in photovoltaic devices. The selected random forest model showed good agreement between predicted and reference LUMO values, indicating its potential for preliminary screening of organic compounds. However, such models require careful interpretation because good interpolation performance within a dataset does not necessarily guarantee reliable extrapolation to chemically distinct molecular structures.

**Figure 4:**
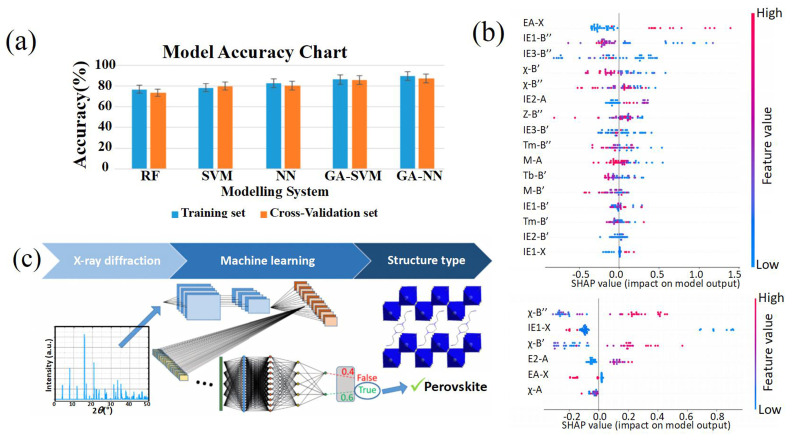
(**a**) The accuracy chart of the different modeling systems. Reprinted from Ref. [72]. (**b**) Scatter plots of feature importance for predicting bandgap and formation energy by the XGBoost Model. Reprinted from Ref. [74]. (**c**) Schematic depicting the screening of structural characteristics and the assessment of feature importance in XRD patterns from both perovskite and non-perovskite materials. Reprinted with permission from Ref. [75]. Copyright 2022 Wiley.

In addition to bandgap and orbital energy levels, ML models have been applied to predict structural stability and screen lead-free or low-toxicity perovskite compositions. Jarin et al. constructed multiple ML algorithms to accelerate the prediction of structural distortions and optimize the comprehensive performance of perovskite materials for renewable energy applications. Basic atomic characteristics of perovskites were adopted as key descriptors to classify crystal structures and forecast lattice parameters [72]. Specifically, ML models, including RF, SVM, NN, and genetic algorithm-assisted neural network (GA-NN), were established for crystal structure classification, while SVR and genetic algorithm-enhanced support vector regression (GA-SVR) models were evaluated for lattice parameter prediction. The GA-NN model achieves an average classification accuracy of nearly 88% for crystal structure identification, and the GA-SVR model delivers a high average prediction accuracy of approximately 95% for lattice constant regression, whose performance can be further enhanced by enriching high-quality datasets, as shown in Figure 4a. These reliable ML models provide valuable insights into the structural behaviors of perovskites and serve as an efficient alternative strategy to accelerate the discovery and development of novel high-performance perovskite materials for renewable energy devices. Similarly, Chen et al. reported that stability-related descriptors and transition-metal features can strongly influence bandgap prediction [73]. Wang et al. used multiple ML algorithms combined with SHAP interpretation to predict the bandgap and formation energy of lead-free double perovskites for photovoltaic applications, as illustrated in Figure 4b [74]. Feature descriptors, including X-site electron affinity, ionization energy, and B/B’’-site electronegativity, were proven to dominate the optoelectronic properties of double perovskites, clarifying the explicit correlation between elemental chemical characteristics and material photovoltaic performance. These investigations demonstrate that descriptor-based ML can effectively bridge intrinsic atomic features and the key properties of perovskite materials. However, such property-prediction models still face limitations, since the prediction performance heavily relies on the quality and scale of existing material databases, and the unexplored chemical space of novel perovskite candidates may cause inevitable prediction errors. This remains a major obstacle to large-scale, accurate discovery of high-efficiency photovoltaic perovskites.

Structural dimensionality and phase identification represent another important application area for ML-guided perovskite analysis. Massuyeau et al. developed ML models, including random forest and convolutional neural networks, to identify structural types of hybrid lead halides from powder X-ray diffraction patterns, as shown in Figure 4c [75]. Their approach enabled automatic classification of unknown compounds and helped distinguish perovskite-type structures with 1D, 2D, and 3D networks constructed from PbX_6_ octahedra. The analysis suggested that Pb–Pb distances and unit-cell volume are important structural features associated with diffraction patterns. This type of ML-assisted structural recognition is valuable because it can accelerate phase identification and reduce the manual effort required for analyzing large experimental or simulated diffraction datasets. The robustness of such models depends on the diversity of training structures, the quality of diffraction data, and the model’s ability to handle peak shifts, preferred orientation, impurities, and mixed phases commonly encountered in experimental samples.

Following the research boom, thousands of papers have been published, providing a sufficient foundation for ML-driven perovskite and PSC applications [76,77,78,79,80]. ML-guided prediction of intrinsic perovskite properties has progressed from simple composition–property regression toward more interpretable, descriptor-rich, and physics-informed models. Bandgap prediction is relatively mature because bandgap datasets are more available and descriptors can be constructed from elemental and structural features. In contrast, predictions of carrier mobility, defect tolerance, phase stability, and long-term degradation remain more challenging because these properties are strongly affected by microstructure, defects, interfaces, processing history, and environmental conditions. Future studies should place greater emphasis on standardized datasets, uncertainty reporting, external test sets, and experimental validation of ML-predicted candidates. Rather than replacing DFT or experiments, ML should be integrated with high-throughput calculations, interpretable descriptors, and closed-loop validation to improve both prediction efficiency and scientific reliability. Building on these intrinsic property predictions, the next section discusses how ML models can be extended from material-level descriptors to device-level performance optimization, where processing parameters, interface engineering, and device architecture become equally important.

Building on these intrinsic property predictions, the next section discusses how ML models can be integrated with high-throughput screening strategies to identify promising perovskite compositions within large chemical spaces.

## 4. High-Throughput Intelligent Screening for Perovskite Composition Optimization

The compositional diversity of perovskite materials provides a vast search space for discovering candidates with suitable bandgaps, structural stability, low toxicity, and favorable optoelectronic properties [81]. By substituting different ions at the A, B, and X sites, a large number of ABX_3_, A_2_BB′X_6_, layered, double, and hybrid perovskite compositions can be generated [82]. Exploring such a high-dimensional compositional space solely through trial-and-error experiments or exhaustive first-principle calculations is inefficient. High-throughput intelligent screening integrates materials databases, physics-based calculations, chemical rules, and ML models to accelerate candidate identification. In this context, ML should be regarded as a complementary tool rather than a direct replacement for DFT, MD, or experimental validation [83]. It can rapidly prioritize promising candidates, while physics-based calculations and experiments remain necessary for mechanistic interpretation and confirmation.

**Figure 5:**
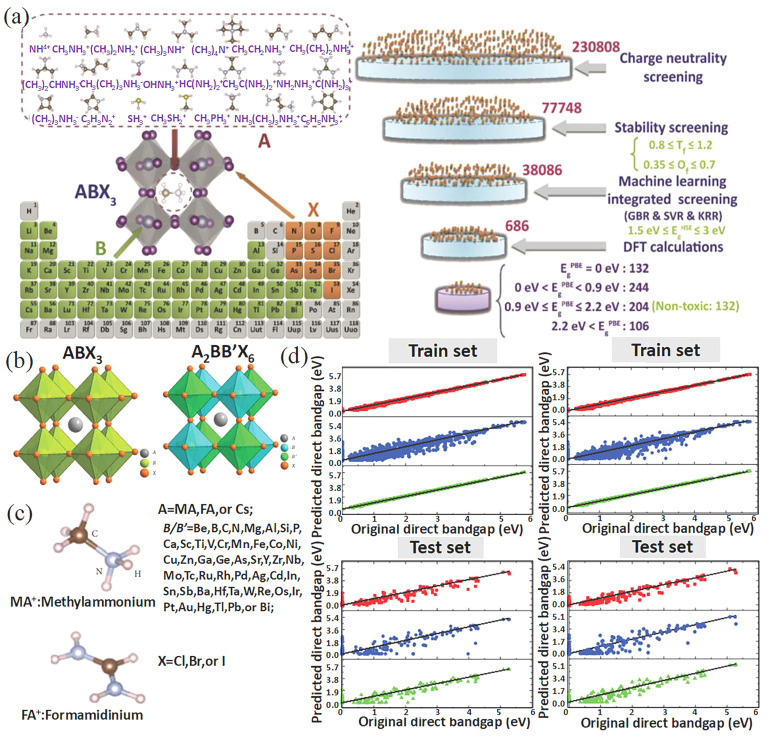
(**a**) Schematic design framework for predicting novel HOIPs based on the combination of ML and DFT calculations for photovoltaic applications. Reprinted from Ref. [87]. (**b**) Perovskite structures in the ABX_3_ and A_2_BB′X_6_. Reprinted from Ref. [91]. (**c**) Two organic molecules MA and FA in perovskite. Reprinted from Ref. [91]. (**d**) The prediction performance of the direct and indirect bandgaps in the train and test sets for HOIPs evaluated using three ML models. Reprinted from Ref. [91].

A reliable high-throughput screening workflow usually begins with the construction of a chemically meaningful candidate space, followed by descriptor generation, preliminary filtering, ML prediction, and validation using DFT or experiments [84,85,86]. Common screening criteria include charge neutrality, tolerance factor, octahedral factor, thermodynamic stability, bandgap range, toxicity, and phase feasibility. Wu and Wang reported a target-driven workflow that combined ML with DFT calculations to screen 230,808 hybrid organic–inorganic perovskites for photovoltaic applications, as shown in Figure 5a [87]. After applying charge-neutrality and stability filters, ensemble learning models, including GBR, SVR, and KRR, were used to predict bandgaps for 38,086 candidates. This process identified 686 orthorhombic-like hybrid perovskites with suitable bandgaps, and DFT calculations further verified 132 stable and Pb-, Cd-, and Hg-free candidates. This study demonstrates how ML can reduce the number of candidates requiring expensive DFT calculations. The screening reliability still depends on the initial chemical rules, descriptor design, and the consistency between ML-predicted and DFT-validated properties.

The development of structured materials databases further supports high-throughput perovskite screening. For example, the JARVIS-Photonics database provides computed optical and electronic properties, including absorption spectra, emission-related properties, bandgaps, and carrier mobility information for a large number of materials [88]. Such databases are valuable because they provide standardized computational data that can be used for model training and benchmarking. In addition, graph-based and structure-aware ML methods have been increasingly used to connect atomic-scale structural features with macroscopic optoelectronic properties [89]. By encoding local bonding environments, coordination geometry, lattice distortion, and electronic descriptors, these methods can capture nonlinear composition–structure–property relationships more effectively than simple empirical rules [90]. Nevertheless, database-derived models may exhibit limited transferability when applied to experimentally synthesized materials, in which defects, disorder, grain boundaries, processing history, and environmental exposure strongly affect measured properties.

ML-assisted screening is particularly useful for exploring large substitution spaces in perovskite compositions. Moeini et al. established a two-dimensional hybrid organic–inorganic perovskite dataset containing structural and bandgap information for 7328 compounds, as shown in Figure 5b,c [91]. The dataset included metal cations such as Pb, Sn, Sb, Cu, Ge, Cd, Bi, Fe, Pd, and Mn, as well as halide anions, including I^−^, Br^−^, and Cl^−^. Regression models, including AdaBoost, decision tree, and GBR, were used to predict bandgap values, while classification models, including gradient boosting machines, decision tree, and multilayer perceptron, were used to distinguish zero-bandgap and nonzero-bandgap compounds, as shown in Figure 5d [91]. Feature analysis indicated that elemental descriptors, such as valence, periodic group, mean values, and standard deviations, play important roles in bandgap determination. This work highlights the potential of descriptor-based ML for rapid bandgap screening. Bandgap prediction can be sensitive to structural dimensionality, spin-orbit coupling, and the source of reference data; external validation using independent DFT calculations or experiments remains important.

**Figure 6:**
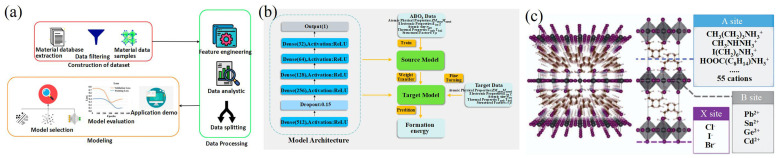
(**a**) The workflow of ML strategy comprised by logistic regression, Multi-layer Perceptron, decision tree, support vector machine, XGBoost, and random forest to optimize the perovskite bandgap. Reprinted from Ref. [92]. (**b**) Overall workflow diagram of the transfer learning strategy for predicting the formation energy of perovskite materials. Reprinted from Ref. [93]. (**c**) The composition of 136 2D HOIPs. Reprinted from Ref. [96].

For classification tasks, Nguyen et al. applied ML to a dataset of 1528 ABX_3_ halide materials extracted from the Materials Project, including compounds containing F, Cl, Br, and Se [92]. Six algorithms, including logistic regression, multilayer perceptron, decision tree, support vector machine, XGBoost, and RF, were evaluated for direct-bandgap classification, as shown in Figure 6a. RF achieved the best performance, with precision of 86%, recall of 85%, and F1 score of 86%. SHAP analysis suggested a relationship between minimum neighbor distance and bandgap behavior, while SMOTE-based data augmentation was used to mitigate class imbalance. This study shows that interpretable classification models can help identify direct-bandgap perovskites for photovoltaic and optoelectronic applications. It should be mentioned that data augmentation cannot fully replace physically diverse training data, and classification performance should ideally be tested on independent compounds not represented in the original dataset.

Transfer learning provides another route to high-throughput screening under limited-data conditions. Yu et al. developed a transfer learning strategy to predict formation energies of doped perovskites [93]. ABO_3_-type perovskites were used as the source domain, while doped perovskite structures were treated as the target domain. A deep neural network was first trained on the source-domain data and then fine-tuned using a smaller number of doped samples, as shown in Figure 6b. The model was used to predict formation energies for more than 72,000 doped compounds, including A_2_BB′O_6_, AA′B_2_O_6_, and AA′BB′O_6_ structures. DFT validation of the lowest-energy candidates confirmed three stable doped perovskites: CaSrHfScO_6_, BaSrHf_2_O_6_, and Ba_2_HfNdO_6_. This example illustrates how transfer learning can improve screening efficiency when target-domain data are scarce, and transferability depends strongly on whether the source and target domains share compatible chemistry, structural representations, and descriptor distributions [94].

High-throughput ML screening has also been applied to impurity and dopant design. Arun et al. combined high-throughput DFT calculations with ML to predict impurity formation energies and charge transition levels in hybrid halide perovskites [95]. Using a dataset of 265 Pb-site impurities across MAPbX_3_ compositions, where X = Cl, Br, I, and mixed halides, they trained Gaussian process regression, random forest, and neural network models using elemental and structural descriptors. Gaussian process regression achieved the lowest prediction errors, within approximately 10% of the target property range. The trained models enabled rapid screening of impurities across multiple perovskite compositions and helped distinguish potentially beneficial dopants for carrier tuning from harmful recombination centers. This work demonstrates the value of combining DFT-generated data with ML for defect-related screening. Impurity behavior in real devices is also affected by defect complexes, grain boundaries, processing conditions, and operating environments, which are difficult to fully capture in simplified computational datasets. Wu et al. applied the GBR algorithm to predict the electronic bandgap of HOIPs [96]. The model was optimized for hyperparameters by a grid search technique and a cross-validation procedure, and 32 features were created to describe the physical and chemical properties of HOIPs. Two hundred nine orthogonal analogs of HOIPs with suitable bandgaps were screened. The component diversity of 136 types of 2D HOIPs in Figure 6c represents the high-dimensional screening challenge that needs to be addressed by machine learning. This provides a diverse set of feature samples for the model and lays a data foundation for the subsequent screening of 2D HOIPs with suitable bandgaps.

Ion migration is a primary factor in the long-term stability of PSC devices, as the translocation of ionic species, particularly iodide, generates defects that hinder charge-transport efficiency [97,98,99]. Quantitative assessment of migration dynamics and critical determinants can be analyzed by tools such as non-negative matrix factorization (NMF) and RF [38,100]. For instance, SHAP-based analysis reveals that surface passivation employing low-polarity materials affects ionic movement, thereby extending the usable lifetime of devices [101,102,103,104,105,106]. Through these ML techniques, it is able to systematically reinforce the durability of perovskite materials, ensuring not only sustained reliability but also establishing a solid basis for eventual industrial-scale deployment.

The key advantage of ML in material screening is the dramatic reduction in time and resources. Especially, the ML-Bayesian strategy quickly narrows down the parameter space, enabling high-throughput virtual screening instead of physically testing among thousands of combinations, which is also transferable to various PSC architectures [107,108,109]. Bayesian strategy offers a fast, cost-effective route to discovering high-performance materials, paving the way for the development of next-generation PSC. It should be emphasized that even though ML has advanced in perovskite screening and design, future efforts should focus on dataset diversity, model interpretability, and real-world data integration to improve device stability predictions. Incorporating chemical rules and stability factors into ML models improves performance, but refining stability criteria and understanding composition relationships remain challenging and integrating real-world experimental data, e.g., degradation patterns, into training models will be the key issue in the future [92].

Overall, high-throughput intelligent screening has become an important strategy for accelerating perovskite composition optimization [110]. ML models can efficiently narrow a large candidate space and identify promising compositions for subsequent DFT calculations or experimental synthesis. The most reliable workflows are those that combine chemical constraints, interpretable descriptors, high-throughput computation, uncertainty-aware ML, and validation beyond the training dataset. Current studies show substantial progress in bandgap prediction, direct-bandgap classification, lead-free candidate screening, formation-energy prediction, and dopant selection. However, several challenges remain unresolved. First, many models are trained on computational databases and may not fully capture experimentally relevant factors, such as defects, disorder, solvent effects, crystallization pathways, and interfacial reactions. Second, stability labels are not standardized and may refer to different criteria, including tolerance factor, formation energy, decomposition energy, phase stability, or operational device stability. Third, model performance is often evaluated using internal train-test splitting, while external validation on newly synthesized materials remains limited. Future high-throughput screening studies should therefore emphasize dataset diversity, uncertainty quantification, out-of-distribution testing, standardized stability criteria, and closed-loop experimental validation. After promising compositions are identified, the next challenge is to translate material-level predictions into device-level performance optimization, where processing parameters, charge-transport layers, interfaces, and device architectures must be considered together.

## 5. ML-Guided Device Optimization, Stability Assessment, and Scalable Manufacturing of PSCs

After promising perovskite compositions, the next challenge is to translate material-level predictions into high-performance, stable, and scalable PSCs [111]. Device performance is not determined by the perovskite absorber alone, but by the coupled effects of absorber composition, crystallization process, charge-transport layers, interface energetics, defect passivation, film morphology, layer thickness, device architecture, and environmental operating conditions [112]. Therefore, ML-guided PSC development has gradually expanded from intrinsic property prediction and composition screening to device-level performance optimization, stability assessment, and commercialization-oriented manufacturing control [113].

### 5.1. ML-Assisted Device Performance Optimization

The performance of PSCs is commonly evaluated using PCE, open-circuit voltage, short-circuit current density, fill factor, hysteresis behavior, and operational stability. These parameters are influenced by nonlinear interactions among material composition, processing conditions, interfacial layers, and device architecture. Conventional experimental optimization is usually based on sequential trial-and-error procedures, which are time-consuming and often difficult to generalize across different material systems. ML provides a data-driven route to capture composition–processing–structure-performance relationships and to identify promising device configurations for subsequent experimental validation [114].

**Figure 7:**
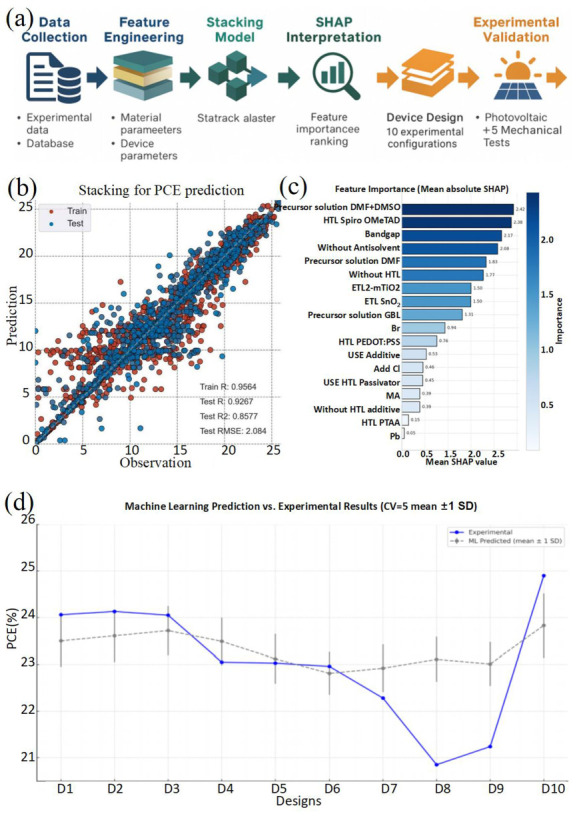
(**a**) Flowchart of machine learning prediction of perovskite solar cells. (**b**) The performance of the Stacking model corrected by experimental data on the training and testing sets. (**c**) Average SHAP values from four machine learning algorithms using one-hot feature encoding, illustrating the top 20 features influencing PCE. (**d**) Comparison between machine learning-predicted and experimentally measured PCE values for ten perovskite solar cell designs (CV = 5 mean ± 1 SD). Reprinted from Ref. [115].

Composition design of PSCs has evolved from single-property bandgap prediction to multi-dimensional optimization covering photovoltaic output and mechanical reliability. Liu et al. constructed a stacking ensemble ML framework coupled with SHAP interpretability for integrated perovskite device design, which simultaneously predicts core photovoltaic metrics, including open-circuit voltage, short-circuit current density, fill factor and PCE, as shown in Figure 7a [115]. The compositional tuning module prioritized A-site FA/Cs cation ratio and precursor solvent systems to screen high-performance mixed-cation perovskite candidates, while the interfacial characteristic design module systematically evaluated the influences of electron/hole transport layer types, dopant additives and anti-solvent treatment on carrier extraction and energy-level matching, as illustrated in Figure 7b,c. The stacked model delivered high predictive accuracy with a test set R^2^ of 0.8577, forecasting optimized device configurations with a peak predicted PCE of 23.82%. Guided by the SHAP feature ranking, ten FA/Cs gradient perovskite devices were fabricated, and the optimal FA_0.85_Cs_0.20_PbI_3_ device achieved an experimental PCE of 24.90%, as shown in Figure 7d. Nevertheless, several limitations still exist. All mechanical characterizations were performed on unencapsulated rigid glass-based devices; thus, the measured mechanical responses are dominated by the glass substrate and fail to reflect the intrinsic mechanical properties of perovskite absorbers and charge-transport layers. The dataset lacks explicit descriptors for crystallization kinetics, ambient humidity, and long-term operational degradation, leading to moderate deviations between predicted and experimental PCE values for partial device configurations. Furthermore, the current framework cannot incorporate long-term operational stability metrics as optimization constraints, restricting its capability to guide the development of commercially viable, durable perovskite photovoltaic modules.

**Figure 8:**
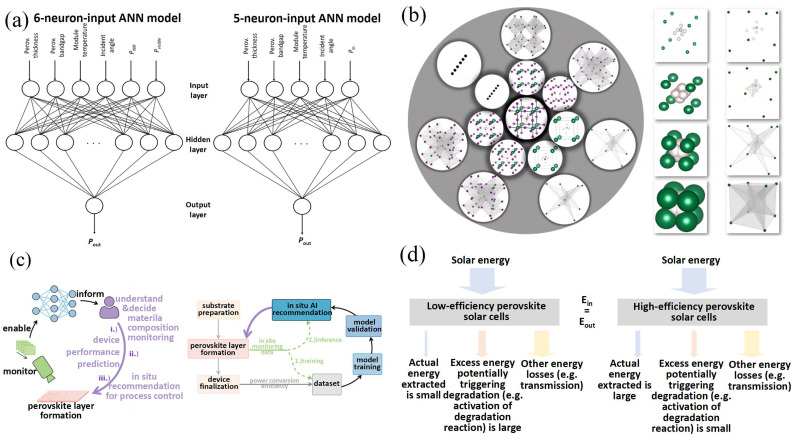
(**a**) Structures of the 5- and 6-neuron-input ANN models. Reprinted from Ref. [116]. (**b**) A multiscale atom-specific representation for OIHP structure. Reprinted from Ref. [122] (**c**) In-situ process metrology augmented by deep learning. Reprinted from Ref. [124]. (**d**) The energy conservation schematic for the PSC system. Reprinted from Ref. [126].

Nguyen et al. constructed an artificial neural network framework combined with Atlas TCAD simulation to predict the annual output energy of two-terminal perovskite/silicon tandem building-integrated photovoltaic cells with inputs covering visible light irradiance, near-infrared irradiance, incident spectral angle, solar module temperature, perovskite bandgap and perovskite thickness, as shown in Figure 8a [116]. Two artificial neural network structures were tested, and the six-input, seventeen-hidden, one-output model achieved best performance with an MSE of 1.26 and a correlation coefficient of 0.99979. The model determined separate optimal perovskite parameters for rooftops and vertical building facades, with the rooftop reaching a maximum annual output energy of 282.54 kWh/m^2^. This ML method greatly reduces massive simulation time and guides building-integrated photovoltaic structural optimization. However, prediction deviations in practical operation may arise from unconsidered factors, such as component aging and varied regional solar spectral conditions.

Li et al. developed an ML framework to predict the PCE of PSCs from device-level and microstructural parameters [117]. Among the evaluated models, including extra trees, k-nearest neighbors, light gradient boosting machine, and RF. The extra trees model achieved the best performance, with R^2^ = 0.71 and MAE = 2.33. Bayesian optimization was further used to search for parameter ranges, such as layer thickness, bandgap, and carrier mobility, that could yield a target PCE. This type of ML-assisted optimization is useful because it can narrow the experimental design space before device fabrication. Nevertheless, an R^2^ value of 0.71 also indicates that a substantial fraction of device performance variation remains unexplained, likely because device performance is strongly affected by hidden variables, such as film morphology, grain boundaries, defect density, interface quality, humidity, and batch-to-batch fabrication variations.

Bayesian optimization, active learning, and surrogate modeling are particularly useful for PSC device optimization because they can reduce the number of required experiments while updating the model as new data are collected. In a closed-loop workflow, initial experimental or simulation data are used to train an ML model, the model recommends new processing or structural parameters, experiments are performed to validate the recommendation, and the new results are fed back into the model. Such closed-loop strategies are more suitable for PSC optimization than one-time prediction models because PSC performance is highly sensitive to processing history and environmental conditions. Future studies should therefore report not only prediction accuracy but also the number of experimental iterations, uncertainty estimates, external validation results, and the reproducibility of the optimized devices.

### 5.2. ML for Scalable Fabrication and In-Line Quality Monitoring

The commercialization of PSCs depends on the successful transition from small-area laboratory devices to large-area perovskite solar modules. However, module-scale PSCs often show lower efficiency than small-area devices because of increased series resistance, nonuniform current collection, film inhomogeneity, pinholes, interfacial defects, and nonuniform crystallization over large substrates [118,119]. As the device area increases, maintaining uniform film thickness, crystallinity, composition distribution, and interface quality becomes increasingly difficult. These challenges make scalable fabrication and quality control central issues for PSC commercialization.

ML-assisted image analysis and in-line metrology provide promising tools for addressing these challenges [120]. In large-area coating or printing processes, optical images, photoluminescence maps, electroluminescence images, hyperspectral images, and other in situ characterization data can be analyzed using ML models to detect defects, monitor crystallization, and predict device performance. Convolutional neural networks and other deep learning (DL) models are particularly suitable for identifying spatially localized defects, including pinholes, cracks, nonuniform grains, and coating irregularities [121]. These methods can potentially enable real-time, non-destructive inspection during perovskite film formation and module fabrication.

Anand et al. proposed a strategy combining multiscale simplicial complexes with persistent functions (PFs), including persistent homology (PH) and persistent Forman Ricci curvature (PFC), to construct intrinsic topological descriptors for halide perovskites [122]. The PF-based feature engineering outperforms traditional material descriptors in phase clustering and bandgap prediction, with comparable accuracy to DL and better interpretability, as shown in Figure 8b. This suits perovskite design as conventional descriptors fail to capture multi-scale atomic interactions. However, the method needs full atomic coordinates and weakens on unseen B/X-site groups, requiring more cross-system validation for real material screening.

Vakharia et al. proposed a strategy combining Multi-Scale SinGAN with heat-transfer search optimization for feature selection from extracted quality parameters [123]. The SinGAN-based ML strategy improved morphology and defect identification and reduced the need for large experimentally labeled datasets. This approach is relevant to PSC manufacturing because labeled defect datasets are often limited, while film morphology can vary substantially with solvent composition, annealing conditions, substrate treatment, and coating method. However, image-based ML models should be validated across different instruments, illumination conditions, substrates, and fabrication batches to ensure robustness in practical production environments.

Laufer et al. demonstrated a deep-learning-assisted approach for monitoring perovskite precursor composition and predicting final device efficiency from early-stage images, as shown in Figure 8c [124]. Such approaches are important because early-stage film-formation features can contain information about crystallization pathways and final film quality. If integrated into coating or printing lines, ML-based monitoring could provide feedback for adjusting processing parameters in real time. Nevertheless, the correlation between early image features and final device performance must be carefully validated, because similar visual morphologies may still lead to different defect densities, interfacial properties, and long-term stability. Therefore, future in-line ML systems should combine image data with complementary measurements, such as photoluminescence, absorption, environmental parameters, and electrical characterization.

### 5.3. ML-Assisted Stability Assessment and Degradation Analysis

Although PSCs have achieved competitive laboratory-scale efficiencies, their operational lifetime remains a major obstacle to commercialization. Stability degradation is affected by perovskite composition, defect density, ion migration, charge-transport layers, interface reactions, electrode diffusion, encapsulation quality, humidity, oxygen, ultraviolet exposure, thermal stress, and electrical bias. Compared with PCE prediction, stability prediction is more challenging because degradation data are heterogeneous, aging protocols are not fully standardized, and degradation mechanisms can vary significantly across device architectures [125].

ML has been applied to large PSC stability datasets to identify degradation patterns and statistical correlations. Hartono et al. collected 2245 maximum power point tracking curves from various device architectures, layers, and perovskite compositions using a high-throughput aging system [126]. The dataset was used to analyze PCE loss after 150 h of aging. Some devices showed an initial light-soaking-induced efficiency increase, whereas others exhibited immediate decay, as shown in Figure 8d. Even though better film quality, lower defect density, improved interface passivation, more favorable charge extraction, or optimized processing conditions can contribute to improved short-term stability, the mechanistic explanation requires controlled experiments, physics-based analysis, and longer-term aging tests. Self-organizing maps and other unsupervised learning methods have also been used to classify degradation curve shapes [126,127]. Such methods can help identify representative degradation modes, including initial gain, exponential decay, linear decay, and complex multi-stage behavior. These classifications are useful because they move beyond single-point stability metrics and allow comparison of degradation trajectories. However, clustering results require appropriate validation metrics and physical interpretation. Without standardized aging protocols, consistent environmental conditions, and metadata describing device architecture and encapsulation, degradation clusters may reflect differences in measurement conditions rather than intrinsic degradation mechanisms.

To improve the reliability of ML-based stability analysis, future studies should standardize degradation datasets by including device structure, active area, encapsulation status, illumination intensity, temperature, humidity, atmosphere, bias condition, aging duration, and measurement protocol [128]. Stability labels should also be clearly defined, for example as degradation rate, normalized PCE loss, or maximum power point tracking lifetime. In addition, uncertainty quantification, external testing, and out-of-distribution evaluation are necessary because stability models trained on short-term aging data may not generalize to long-term outdoor operation or module-level degradation.

### 5.4. Role of Physics-Based Simulations in ML-Guided PSC Design

Physics-based simulations remain important for understanding device behavior and validating ML predictions. Finite element modeling, drift-diffusion simulation, optical simulation, and Poisson equation-based device modeling can provide insights into charge transport, recombination, optical absorption, electric-field distribution, and interface effects [129,130]. ML can complement these methods by constructing surrogate models from simulation data, reducing computational cost during parameter search, and identifying promising regions of the design space [129]. Conversely, physics-based models can constrain ML predictions and improve interpretability. Plasmonic or nanostructure-enhanced PSC designs may show high simulated PCE values under optimized optical and electrical assumptions, but such values do not necessarily represent experimentally realized device efficiencies. The practical implementation of these structures may face challenges related to fabrication complexity, parasitic absorption, long-term stability, and compatibility with scalable manufacturing. Therefore, simulation-based studies are most valuable in an ML-guided review when they either provide training data, physical constraints, or validation benchmarks for ML models.

### 5.5. Commercialization-Oriented Perspective

For PSC commercialization, the most relevant opportunities include large-area film uniformity control, defect detection, precursor and coating optimization, module-level performance prediction, encapsulation reliability, degradation monitoring, lead leakage risk assessment, and closed-loop process control [131,132,133]. Table 2 shows the recent studies on ML applications to perovskite materials and their PSC applications in detail [134,135,136,137,138,139,140,141,142,143,144,145,146]. Overall, ML-guided device optimization and manufacturing control can accelerate the transition of PSCs from laboratory-scale devices to scalable modules. The most reliable approaches are those that combine experimental datasets, in situ characterization, physics-based simulation, uncertainty-aware ML, and closed-loop validation. At the same time, current models remain limited by small and heterogeneous datasets, insufficient external validation, unclear distinction between predicted and certified performance, and the lack of standardized stability protocols. Future work should focus on reproducible workflows, module-level validation, long-term aging data, interpretable models, and integration of ML with automated fabrication and real-time process monitoring. These device-manufacturing-level studies show the practical potential of ML-assisted PSC development, but they also reveal unresolved challenges related to data standardization, model reliability, long-term stability, and experimental validation, which are discussed in the final section.

## 6. Challenges, Perspectives and Conclusions

ML has become an increasingly important tool for accelerating perovskite material discovery and perovskite solar-cell development. As discussed above, ML has been applied to intrinsic property prediction, high-throughput composition screening, device performance optimization, stability assessment, and manufacturing-related monitoring [147]. These studies demonstrate that ML can reduce the experimental and computational search space, identify important descriptors, guide candidate selection, and support closed-loop optimization. However, the current state of ML-driven PSC research should be viewed with appropriate caution. Many reported models are still based on limited, heterogeneous, or computationally generated datasets, and large-scale experimental verification remains uncommon. Therefore, future progress will depend not only on developing more advanced algorithms, but also on improving data quality, validation standards, model interpretability, and experimental integration.

### 6.1. Data Infrastructure and Standardization

The reliability of ML models is fundamentally limited by the quality and representativeness of the underlying data. In PSC research, data are often collected from different laboratories, device architectures, measurement protocols, environmental conditions, and reporting standards. As a result, nominally similar parameters, such as PCE, stability lifetime, degradation rate, or bandgap, may not be directly comparable across studies. This issue is particularly severe for stability-related data because aging tests may differ in illumination intensity, temperature, humidity, atmosphere, encapsulation, bias condition, and measurement duration.

Future ML-driven PSC research requires standardized and machine-readable datasets that include both target values and complete metadata [50]. For material-level prediction, datasets should report composition, crystal structure, dimensionality, synthesis route, measurement method, and uncertainty. For device-level prediction, datasets should include device architecture, layer thickness, transport materials, active area, processing parameters, annealing conditions, encapsulation status, and testing protocols. For stability prediction, normalized metrics, such as maximum power point tracking lifetime, degradation rate, and normalized PCE loss, should be reported together with environmental and operational conditions. FAIR data principles, open databases, and benchmark datasets will be essential for meaningful comparison among ML models.

### 6.2. Model Reliability, Uncertainty, and Generalization

High internal prediction accuracy does not necessarily imply reliable performance on new perovskite compositions, device architectures, or processing conditions [148,149]. Many existing ML studies rely on random train–test splitting, which can overestimate model performance when chemically similar samples appear in both training and test sets. Data leakage, small datasets, class imbalance, and biased literature-derived data can further reduce the reliability of reported results. Therefore, future studies should include external test sets, out-of-distribution evaluation, uncertainty quantification, and clear reporting of model applicability domains.

Model interpretability is also crucial for scientific understanding. Methods such as SHAP analysis, feature importance ranking, and physically informed descriptors can help identify key factors controlling bandgap, stability, defect formation, and device performance. Statistical correlations must be validated using independent experiments, physics-based simulations, or mechanistic analysis. Reliable ML models should not only predict target properties, but also indicate when their predictions are uncertain or outside the training domain.

### 6.3. Integration with Physics-Based Simulations and Experiments

ML should be integrated with, rather than treated as a replacement for, physics-based calculations and experimental validation. DFT, MD, Monte Carlo simulations, finite element modeling, and drift-diffusion simulations remain essential for understanding electronic structure, defect physics, ion migration, charge transport, optical absorption, and interface behavior. ML can accelerate these methods by constructing surrogate models, prioritizing candidates for high-cost calculations, and guiding experimental design. Conversely, physics-based simulations can provide physically meaningful training data, constraints, and validation benchmarks for ML models.

The most reliable workflow for ML-driven PSC research is a closed-loop framework that combines prediction, validation, and feedback. In such a workflow, ML models first identify promising compositions, processing conditions, or device structures [150]. DFT calculations, device simulations, or experiments then validate the predictions. The new results are fed back into the dataset to improve subsequent model performance. This iterative strategy is particularly important for PSCs because material performance is strongly affected by hidden variables, such as crystallization pathway, defect distribution, interface chemistry, and processing history.

### 6.4. Stability-Oriented and Manufacturability-Oriented Prediction

Although ML has shown strong potential in predicting bandgaps, formation energies, and other intrinsic properties, predicting long-term operational stability remains substantially more difficult. PSC degradation involves coupled effects of moisture, oxygen, ultraviolet exposure, thermal stress, electrical bias, ion migration, interfacial reactions, electrode diffusion, and encapsulation failure [151,152,153,154]. These degradation pathways are highly dependent on device architecture and operating environment. Therefore, stability models trained on short-term or narrowly defined datasets may not generalize to long-term outdoor operation or module-level reliability [155,156].

Future ML models should move beyond efficiency-centered prediction and incorporate stability, reproducibility, scalability, and environmental robustness as key optimization targets [157,158,159,160,161,162]. In addition to laboratory-scale PCE, important outputs should include degradation rate, lifetime, thermal stability, humidity tolerance, UV stability, hysteresis, module efficiency, film uniformity, defect density, encapsulation reliability, and lead leakage risk. Multi-objective optimization will be essential because high PCE alone does not guarantee long lifetime or manufacturability.

### 6.5. Autonomous Platforms and Closed-Loop Manufacturing

The integration of ML with automated synthesis, high-throughput characterization, in situ imaging, and robotic experimentation represents an important future direction for PSC development. Autonomous platforms and self-driving laboratories can iteratively select experiments, synthesize materials, characterize films or devices, update ML models, and optimize objectives, such as PCE, stability, processing window, or film uniformity [163,164,165,166,167,168,169]. Such platforms are particularly valuable for PSCs because the final device performance is sensitive to precursor chemistry, solvent evaporation, crystallization kinetics, annealing conditions, and ambient environment.

For commercialization, ML should also be embedded into scalable manufacturing workflows. Real-time monitoring of blade coating, slot-die coating, printing, annealing, and encapsulation processes can provide feedback for process control. Image-based DL, hyperspectral imaging, photoluminescence mapping, and electroluminescence analysis can help detect defects, predict device performance, and improve module consistency. However, these systems require robust models that can operate across different instruments, production lines, environmental conditions, and material batches.

In summary, ML has significantly expanded the toolbox for perovskite materials and PSC research. It has enabled rapid property prediction, high-throughput composition screening, device-performance optimization, degradation analysis, and manufacturing-related quality control. Nevertheless, the field is still transitioning from proof-of-concept prediction toward reliable, experimentally validated, and industrially relevant ML workflows. The major challenges include limited and heterogeneous datasets, insufficient external validation, unclear reporting of uncertainty, weak extrapolation ability, inconsistent stability metrics, and limited integration with real-world manufacturing data. Future progress will require a shift from algorithm-centered studies to reliability-centered and application-driven ML frameworks. Standardized datasets, transparent benchmarks, uncertainty-aware models, interpretable descriptors, external validation, closed-loop experimentation, and module-level stability testing should become routine practices. By combining ML with physics-based simulations, automated experimentation, in situ characterization, and scalable manufacturing control, reliable ML-driven strategies can accelerate the development of efficient, stable, and commercially viable perovskite solar cells.

## Figures and Tables

**Table 2:** Overview of recent studies on ML applications on perovskite materials and its PSC applications.

Objective	Year	ML Technique	Results	Ref.
Device performance optimization	2025	SHAP, Random Forest Regression	SCAPS-1D simulation combined with ML was adopted to design and optimize Cs_2_RbGaI_6_ double perovskite solar cells. The ML-predicted simulated PCE reaches 22.56%, with R^2^ = 0.9985.	[134]
	2026	Random forest Regression	Lead-free Al/FTO/TiO_2_/Ba_3_SbI_3_/CuSbS_2_/Au perovskite solar cell was optimized via simulation and random forest regression. All photovoltaic parameters below are ML-predicted simulated values reach PCE = 32.91%, open-circuit voltage (V_o_c) = 1.18 V, short-circuit current density (J_s_c) = 32.16 mA/cm^2^, fill factor (FF) = 86.38%. Model predictive accuracy R^2^ > 0.92.	[135]
	2026	Random Forest Regression	A novel lead-free dual-absorber perovskite architecture Al/FTO/SnS_2_/Ca_3_SbI_3_/Ba_3_SbI_3_/CBTS/Au was designed. ML-predicted simulated results reach PCE = 36.03%, J_s_c = 32.18 mA/cm^2^, V_o_c = 1.305 V, FF = 85.84%. Model statistical metrics: average R^2^ = 0.9635, MAE = 0.4506, RMSE = 0.6253.	[136]
Scalable fabrication and in-line quality monitoring	2024	DL	DL combined with explainable artificial intelligence(XAI) tackles unstable quality issues hindering large-area perovskite film commercialization, replacing inefficient trial-and-error fabrication tuning. This work constructs interpretable links between real-time film-forming sensor data and cell performance, extracts practical processing strategies for industrial manufacturing, and validates XAI’s key value in accelerating energy material research.	[137]
	2025	Multimodal convolutional neural network	The multimodal CNN extracts microstructural features from experimental perovskite SEM images and weights multimodal information dynamically to elevate prediction accuracy. Trained on 1583 experimental SEM image datasets, the model achieves R^2^ = 0.79. Two grain-related experimental thresholds strongly correlate with PCE improvement, grain boundary length density < 5.96, equivalent circular diameter > 0.83.	[138]
	2026	RF, XGBoost, MLP, SVR, KNN	Random forest delivers the highest prediction accuracy with R^2^ close to 0.8. The model can screen over 100,000 processing parameter combinations in 10 min, which equals 3 years of manual experimental trial-and-error work for scalable perovskite film manufacturing. All predicted parameters correspond to experimental fabrication routes.	[139]
Stability assessment and degradation analysis	2025	Squeeze-and-excitation residual network, SHapley Additive exPlanations,	A multihead attention-integrated SEResNet model processes experimental internal and external aging parameters simultaneously. For experimental device stability prediction, the model reaches R^2^ = 0.972 and Pearson correlation r = 0.986. High-throughput screening of ~2000 experimental device samples quantifies interactions between core stability factors and outputs the optimal architecture stable under 85 °C/85% RH accelerated aging test conditions.	[140]
	2025	Modified Grasshopper Optimisation Algorithm, entropy-based support vector machine(ESVM)	The framework normalizes and augments experimental stability datasets, removes redundant features via MGO, and classifies experimental PSCs into stable/unstable categories using ESVM. The proposed method attains a classification accuracy of 0.99, outperforming benchmark algorithms; all labels are derived from experimental aging data.	[141]
	2025	RF, XGBoost	Models were trained on the open experimental Perovskite Database. On test experimental data, stability classification accuracy = 0.848 and PCE prediction R^2^ = 0.751. Stability criterion is defined as retaining ≥80% of initial experimental PCE after 1000 h continuous operation. Trained models conduct high-throughput virtual screening of 29,016 simulated novel device stacks with varied transport/absorber layers and ion compositions; 100 optimal stable lead-based PSC configurations are filtered, with a maximum ML-predicted simulated PCE up to 26.06%.	[142]
ML-guided PSC design	2026	Linear Regression, K-Nearest Neighbors, RF, XGBoost	The device stack uses BixA_1−x_FeyB_1−_ᵧO_3_ as the absorber, ZnO as ETL and CuSCN as HTL. Model performance: R^2^ = 1.00000, RMSE = 0.00002. After ML optimization of absorber stoichiometry (BSFCO-10, Sm-10%, Co-10%), the ML-predicted simulated PCE of the optimal device reaches 14.08%.	[143]
	2026	RF, SVR, Neural Networks, XGBoost	XGBoost exhibits the best predictive capacity with R^2^ = 99.99% and MSE = 0.003, precisely forecasting simulated photovoltaic performance and identifying dominant governing parameters. For the optimized Au/MoO_3_/Rb_2_LiGaI_6_/WO_3_/FTO lead-free double perovskite architecture, ML-predicted simulated values for photovoltaic outputs are PCE = 32.64%, V_o_c = 1.00 V, J_s_c = 38.02 mA/cm^2^, FF = 85.85%.	[144]
	2026	XGBoost	SCAPS-1D simulation coupled with XGBoost optimizes WS_2_/Zn_3_P_2_ modified MASnI_3_ lead-free perovskite solar cells, yielding an optimized ML-predicted simulated PCE of 32.30%. XGBoost achieves ultrahigh prediction accuracy (R^2^ = 0.9999). Feature importance ranking identifies defect density as the primary factor limiting simulated cell efficiency.	[145]
	2026	XGBoost, RF, Gradient Boosting	WS_2_/CuI transport layers are integrated into RbGeI_3_ lead-free perovskite devices. The maximum ML-predicted simulated PCE of the optimized stack is 24.15%. All three ML algorithms deliver R^2^ approaching 1.0, and XGBoost extracts the most critical structural/material parameters dominating simulated device performance.	[146]

## Data Availability

No new data were created or analyzed in this study. Data sharing is not applicable to this article.

## Abbreviations

The following abbreviations are used in this manuscript:

PSC	Perovskite solar cell
ML	Machine Learning
AI	Artificial intelligence
PCE	Power conversion efficiency
RF	Random Forest
DFT	Density functional theory
MD	Molecular dynamics
KRR	Kernel Ridge Regression
SHAP	SHapley additive explanation
MLP	Multilayer perceptron
DL	Deep learning
